# C3PO: a crop planning and production process ontology and knowledge graph

**DOI:** 10.3389/frai.2023.1187090

**Published:** 2023-10-16

**Authors:** Baptiste Darnala, Florence Amardeilh, Catherine Roussey, Konstantin Todorov, Clément Jonquet

**Affiliations:** ^1^LIRMM, CNRS, University of Montpellier, Montpellier, France; ^2^Elzeard, Bordeaux, France; ^3^MISTEA, INRAE, Institut Agro, University of Montpellier, Montpellier, France

**Keywords:** ontology, semantic resource, knowledge representation, knowledge graph, agriculture, crop planning, crop production process, digital farming

## Abstract

Vegetable crop farmers diversify their production by growing a range of crops during the season on the same plot. Crop diversification and rotation enables farmers to increase their income and crop yields while enhancing their farm sustainability against climatic events and pest attacks. Farmers must plan their agricultural work per year and over successive years. Planning decisions are made on the basis of their experience regarding previous plans. For the purpose of assisting farmers in planning decisions and monitoring, we developed the *Crop Planning and Production Process Ontology* (C3PO), i.e., a representation of agricultural knowledge and data for diversified crop production. C3PO is composed of eight modules to capture all crop production dimensions and complexity for representing farming practices and constraints. It encodes agricultural processes and farm plot organization and captures common agricultural knowledge. C3PO introduces a representation of technical itineraries, i.e., sequences of technical farming tasks to grow vegetables, from soil identification and seed selection to harvest and storage. C3PO is the backbone of a knowledge graph which aggregates data from heterogeneous related semantic resources, e.g., organism taxonomies, chemicals, reference crop listings, or development stages. C3PO and its knowledge graph are used by the Elzeard enterprise to develop knowledge-based decision support systems for farmers. This article describes how we built C3PO and its knowledge graph—which are both publicly available—and briefly outlines their applications.

## 1. Introduction

Agricultural work is complex, farmers need to take various factors such as weather, seasonality, commercial demand, and plant life cycles into account when planning production. Moreover, consumers' and farmers' behavior and practices are changing, with greater consideration for ecological and economic aspects. After the WWII, single-crop farming and the use of chemical inputs were highly promoted, but this led to reduced soil fertility and chemical contamination of soil and crops. In recent years, farmers have begun adopting agroecological practices. Agroecology offers a way of designing farming production systems that rely on agroecosystem functionalities. Crop diversity is a key aspect of this approach. Several scientific studies (Isbell et al., 2017; Paut et al., 2019) have shown that, in both spatial and temporal terms, crop plant diversity (i) improves risk management under changing weather and economic conditions; (ii) delivers a natural defense system against diseases and pest attacks; and (iii) increases agroecosystem stability and resilience. Farmers must consider different stages of development, treatments, and biological interactions between plants. However, these new agroecological practices increase farmers' workload, management complexity, and mental burden (Morel and Léger, 2015; Dumont, 2021). Between 2019 and 2020, the SME Elzeard conducted over 150 interviews with vegetable farmers, agricultural advisors, teachers, and researchers.^1^ Elzeard identified several technological barriers, including the need for knowledge sharing and new operational tools to assist vegetable farmers in their daily crop management, i.e., optimizing crop rotations, yields, and finding alternatives to chemical inputs.

Agricultural knowledge–i.e., consensual information used by farmers to make decisions and to take actions–is currently scattered webwide, in books, archives, and databases while also conveyed informally through interpersonal communication and cultural practices. As well, climate change is increasing an important factor to mitigate in agriculture. Climate has a direct impact, with rainfall fluctuations and heat waves, affecting plant growth and promoting disease emergence (Mendelsohn, 2009; Arora, 2019). To help farmers face this new challenge and its impact on agriculture, it is necessary to share and discover new knowledge. Climate change and the adoption of agroecology approaches now call for the development of novel knowledge-based systems. Knowledge needs to be formalized and shared with anyone who needs it, requiring a common formalization. Moreover, quality knowledge must be readily findable, accessible, interoperable, and reusable (FAIR) for users (Wilkinson et al., 2016). In that respect, semantic web technologies facilitate the development of robust knowledge-based systems by enabling formalization and sharing of knowledge for validation, enrichment, and discovery with native reasoning. Common formalization also facilitates the aggregation of diverse resources and provenance verification to control the quality of shared knowledge.

Semantic Web technologies are used to build knowledge graphs (KG) and ontologies in agriculture (crops and livestock farming, etc.) for both experimental and agricultural purposes (Drury et al., 2019). For instance, AgroPortal (Jonquet et al., 2018) hosts approximately 150 ontologies and vocabularies (as of January 2023), many of which focus on agrifood-environment issues, such as the Agronomy ontology (Devare et al., 2016) and FoodOn (Dooley et al., 2018). However, diversified vegetable farming has not been extensively explored, and there is a substantial need for research in this area. Diversified vegetable crop farmers define technical itineraries (“*itinéraire cultural”* in French, abbreviated in the domain as ITK) as sequences of technical farming tasks to grow vegetables from soil identification and seed selection to harvest and storage. In these sequences, every task and their timing depend on each other and other parameters such as the cropping mode (open field or under cover) or the climatic conditions. As an illustration, planting an onion crop in winter can lead to a spring harvest if cultivated under cover. However, if the onions are cropped in an open field, they will be harvested in the summer. Farmers and agricultural experts define the technical itineraries and draw up plans for the following year(s) based on what happened previously in the fields. They can share their technical itineraries with other farmers who will adapt them to their specific agricultural context, as defined by parameters such as soil type and climate, the number of farm workers or the diversity of crops. To the best of our knowledge, technical itineraries have not been represented using Semantic Web technologies.

In this study, we developed the *Crop Planning and Production Process Ontology* (C3PO) and populated it into a KG. The couple jointly captures vegetable crop farming management concepts and knowledge to support multiple applications in diversified crop planning. The ontology incorporates the representation of technical itineraries for farm planning and management, as well as the representation of plants, plot organization, and chemical products and equipment. The KG is aligned with other agricultural Semantic Web resources to integrate reference data dispersed in organization systems. Our goal is to represent interactions between living organisms, farmers' actions, and input products. Each type of entity is identified and managed under different web standards. This study presents the methodology by which the ontology and related KG have been developed. A subpart of the C3PO knowledge graph will soon be publicly available on *La Serre des Savoirs*, a web portal that pools integrated and harmonized knowledge about plants and farming practices. In addition to these knowledge assets, we are building multiple applications such as two knowledge-based decision support systems to assist farmers: *Elzeard*, a web and mobile application to plan and monitor crop production in vegetable farming systems; and *La Pépinière*, a free web application to help beginners to design their farms and their future production system.

The rest of the study is organized as follows: Section 2 presents related work in ontology development methodology, agriculture, and semantics; Section 3 outlines the methodology implemented to build the ontology and knowledge graph; Section 4 presents the ontology and knowledge graph; Section 5 presents applications based on the ontology and knowledge graph; Section 6 discusses the problems encountered and limitations of our study; and Section 7 concludes and presents the perspectives.

## 2. Related work

Many ontology and knowledge graph development methodologies have been created to support ontology development since the 1990s. In this section, we present those used in the development of our resource. The NeOn methodology (Suárez-Figueroa et al., 2012) presents nine flexible scenarios to build an ontology and “ontology networks” based on the reuse of semantic resources, the transformation of non-semantic resources, and the reuse of ontology design patterns. An ontology network is a collection of ontologies linked *via* relations such as mapping, import, or version. By this methodology, we designed C3PO in multiple ontology modules to address different scenarios. The list of C3PO's modules is presented in Section 4.1. According to the NeOn methodology definition, an ontology module is “a part of the ontology that defines a relevant set of terms”. However, although NeOn offers interesting guidelines to organize the ontology development, the methodology is time-consuming due to the required quantity of documentation and the NeOn toolkit, i.e., the integrated development environment proposed with the methodology, is no longer updated. The Agile methodology was originally used for the development of systems and applications, then later also for ontologies, which implies iterative development and publication and continuous collaboration with consumers. SAMOD (Peroni, 2016) is an Agile methodology with which ontologists develop small ontology iterations for describing a particular use-case and addressing competency questions. After review, the iteration is added to the main ontology representing the whole domain. We adopted this approach for the development of each C3PO module. The Linked Open Terms (LOT) methodology (Poveda-Villalón et al., 2022) is another Agile methodology describing each ontology development step, from specification to publication. LOT is focused on industrial projects as the aim to be compatible with software development methodologies with iterative steps. Moreover, a set of tools is provided as well as examples of how they may be used in ontology development. We built C3PO by combining the development steps presented in LOT and the iteration development process presented in SAMOD.

We studied models focused on agronomy and agriculture. We queried AgroPortal to identify ontologies and vocabularies to represent plant knowledge, agricultural tasks, and plot organization and identified multiple semantic resources: the French Crop Usage thesaurus (FCU) (Roussey, 2018), a list of cultivated plants organized by agriculture uses in France; the Agroecology Knowledge Management application (GECO, in French), a research information system for the GECO data graph (Soulignac et al., 2019) to design innovate agroecology-oriented crop systems; TAXREF-LD (Michel et al., 2017), a linked data representation of the national repository of fauna and flora of France; the NCBI Taxonomy, a curated classification and nomenclature for organisms; Plant Ontology (Jaiswal et al., 2005), a structured vocabulary and database resource that links plant anatomy, morphology, growth, and development to plant genomics data; Crop Ontology (Arnaud et al., 2012), a vocabulary of observable characteristics of common crops for food and agriculture; and the AgroLD knowledge graph focused on plant biology data (Larmande and Todorov, 2021).^2^ Each of these resources is based on a specific viewpoint but cannot be used alone to represent plant knowledge in agriculture. However, we have combined some of them in a coherent integrated knowledge graph that is presented later. Other ontologies and vocabularies available in AgroPortal-but which we did not directly used in our work- to represent agricultural processes include: the Agronomy Ontology (AGRO) (Devare et al., 2016), which represents agronomic experiments by recording precise observations concerning experiments on agricultural plots but is not geared toward agricultural planning and monitoring. The DEMETER Agriculture Information Model (Palma et al., 2022) focuses on smart farming solutions using sensors to monitor crops, which is currently beyond our scope.

Semantic Web technologies may represent processes which could be used to represent tasks in agriculture. The Provenance Ontology (Prov-O) (Lebo et al., 2013) traces the provenance and evolution of activities, interacting with involving agents and entities. Prov-O is an interesting ontology that needs to be specialized to represent a domain, but it does not address all of our needs, especially with respect to temporal aspects required for representing technical itineraries. Otherwise, it is essential to represent theoretical dates, i.e., dates not related to a year (e.g. 04/25), which is not possible. We, thus, opted to use the Time Ontology (Hobbs and Pan, 2006) and extended it to fulfill our needs. However, Prov-O is used to track KG updates, as explained in Section 3.4. We also studied ValueFlows, an ontology that describes economic value flows according to three representation layers (Knowledge, Plan, and Observation).^3^ Knowledge represents plan specification to make something: an ordering set of tasks (e.g., a cooking recipe which specify step-by-step the recommended quantity of ingredients to cook); plan represents the planning of these tasks by an agent and the choice made to implement them (e.g., the actual recipe steps with the quantity of ingredients planned to be used); observation represents the plan execution of the tasks (e.g., the actual recipe steps followed by the cook with the quantities of ingredients used). We used these three layers to conceptualize the principles underlying the representation of technical itineraries: plan specification, plan, and plan execution.

To the best of our knowledge, there are currently no ontology available to help diversified vegetable crop farms in planning and managing their farming tasks. Existing ontologies either only represent sub-parts of the problem or focus on other agricultural sectors, such as cereal cropping or livestock farming. However, the representation of technical itineraries for diversified vegetable agriculture and their use for agricultural planning has not been addressed in an adequate and complete ontology.

## 3. Requirements and methodology for FAIR ontology building and sharing

**Figure 1:**
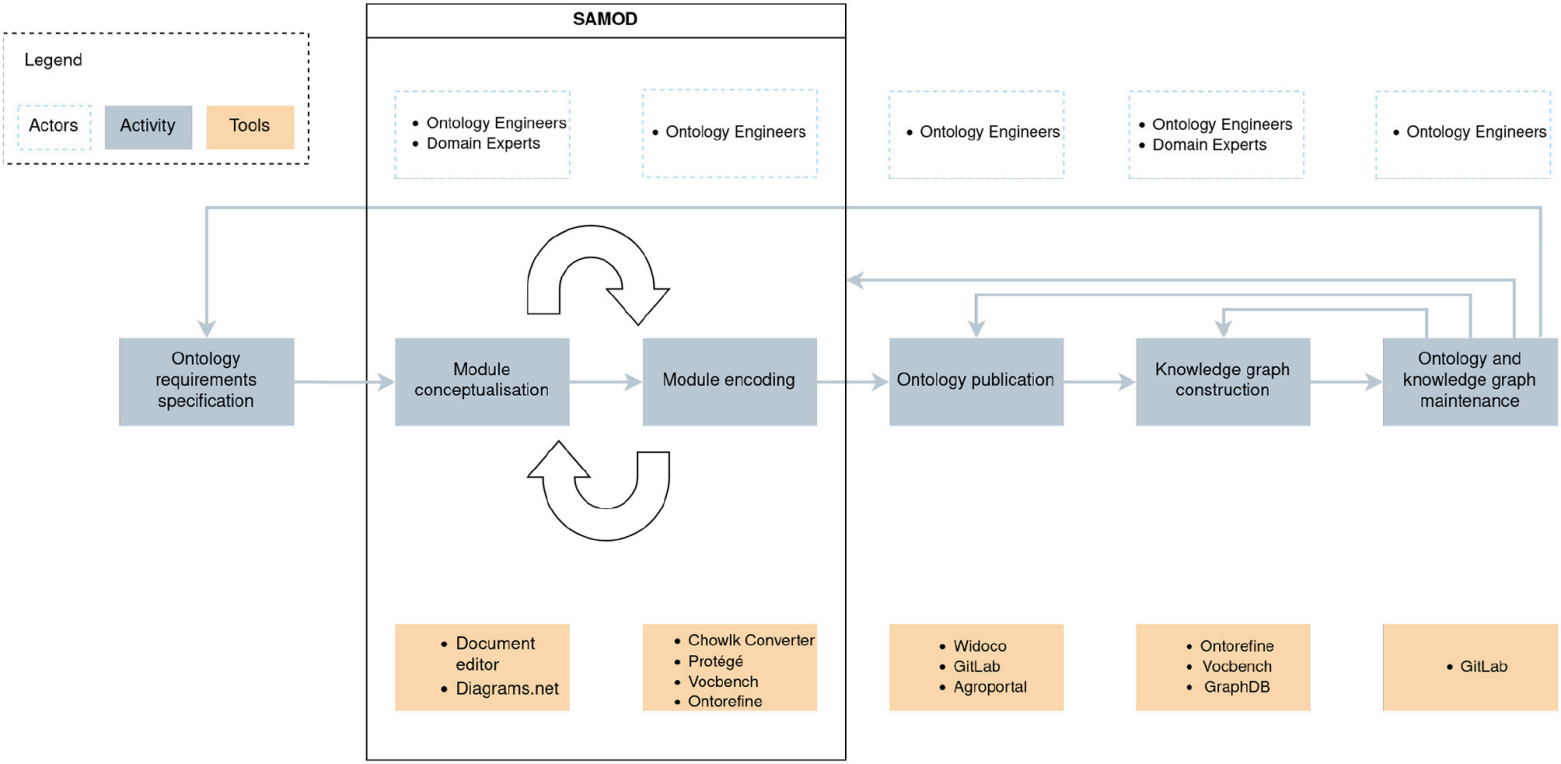
C3PO implementation steps in the LOT/SAMOD methodologies and tools used.

As mentioned previously, we built C3PO using a combination of LOT and SAMOD. We followed the LOT workflow, consisting of the ontology: (i) requirement specification, (ii) implementation, (iii) publication, and (iv) maintenance. The methodology also includes the knowledge graph construction and maintenance procedures. In the development, we built a component that meets current needs before adding it to the ontology, as recommended by SAMOD. Combining these methodologies brought us: (i) a general process regarding the construction of the ontology, due to LOT; (ii) a process regarding the update of the ontology though iterative development, thanks to SAMOD. We recommend this combination to any ontology development project related to an application development using agile methodology, with multiple viewpoints, multiple subdomains, and that integrate several and heterogeneous data sources. The main actors are the Domain Expert (DE), i.e., who offers the domain knowledge covered by the ontology and the overall vision of the work, and the Ontology Expert (OE), who has expertise in ontology and knowledge representation methods. Figure 1 presents the C3PO implementation steps, people involved, and tools used.

### 3.1. Ontology requirement specification

#### 3.1.1. Use-case specification

This specification involves collecting the requirements for the ontology. The DEs present the needs in terms of data and queries required with informal text and schemas. This study defines the scope of the domain of the ontology should address and specify the use-cases. Moreover, DEs and OEs can extract multiple sub-domains for the ontology, which leads to the creation of several ontology modules. OEs review the document to gain insight into the data need, constraints, and logical inference expected and split the use-case regarding the different domains. Moreover, OEs also distinguish between static qualitative data (e.g., in our case plant families, inputs, crops, physical locations, and laws) and user input data. This difference is important as the implementation needs are not the same: user data will be collected *via* forms or sensors, then curated and eventually analyzed, whereas qualitative data that are relevant for the domain will have to be found by OEs in relevant external knowledge sources in the form of open data, existing ontologies or KGs, community standards, and norms. When such standards do not exist, OEs will have to build them. For instance, the way farmers organize their plots is typically user data, while the taxonomic representation of plants represented in TAXREF-LD is a relevant knowledge source that has been integrated into the C3PO knowledge graph.

#### 3.1.2. Functional ontological requirements

OEs describe each use-case with a title, an informal description, and competency questions related to this use-case to support the ontology development process. Examples of use-case and some competency questions for C3PO are presented in Table 1. Some competency questions are also described in the ontology metadata and in the documentation.^4^

**Table 1:** Use-case description of the representation of plants and their families.

**Title**	**Representation of the organization of plants and their groups**
Description	Plants are organized in several families (group of plants that share some common characteristics). These families can describe botanical characteristics, or can describe their usage in agriculture or consumption. Representing only the plant and the families is not sufficient as plants have cultivars that could be split in multiple categories (called varietal types) regarding their physical characteristics. For example, onion can be divided into yellow onion and red onion. The need is to get a representation of the whole plant taxonomy to enhance the farmers' knowledge about plant characteristics.
Competency Questions	1. What are the botanical and usage families of a given crop? Botanical family of the onion is amaryllidaceae and usage family is bulb vegetables. 2. Are these two given crops from the same family? Onion and garlic are from the same botanical family. 3. What are the crops in a given botanical or usage family? Onion, garlic, and shallot are in the amaryllidaceae botanical family.

### 3.2. Ontology implementation

#### 3.2.1. Ontology conceptualization

To conceptualize an ontology module that fulfills the requirements, we opted to use the SAMOD methodology because it offers the possibility to build an ontology module for some use-cases before integration in the main ontology. The proposition at the end of the conception phase is a list of concepts, relations, and queries.

**Figure 2:**
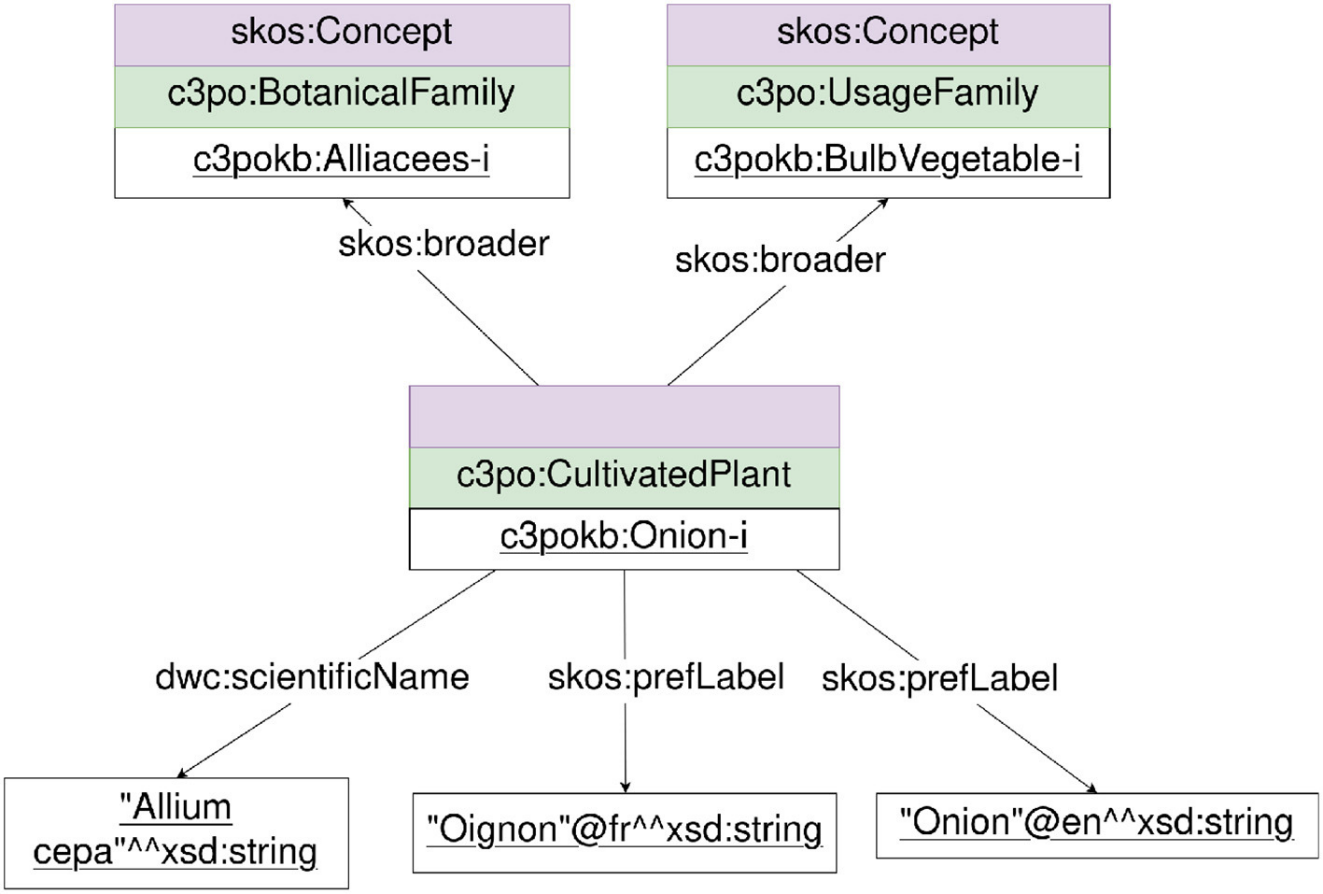
Representation of plants and their families.

OEs analyze the requirements and extract a list of concepts and relations. They propose a name, URI, and definition for each one. As an example of the use-case in Table 1, an extractable concept is a “crop”, however we choose to create the “CultivatedPlant” class with an URI and associated definition: “Vegetal organism cultivated by human beings”. Then, an ontology module is built with the classes and properties proposed with Chowlk notation (Chávez-Feria et al., 2022), i.e., an UML-based notation to build ontology diagrams in Diagrams.net.^5^ The diagram is composed of the classes, properties, and an instantiated data example. An example of a diagram produced and related to the example in Table 1 is presented in Figure 2, this is a representation of the onion, its families, and labels. The individuals of the plants and their families are both typed skos:Concept and owl:Class.

#### 3.2.2. Ontology conceptualization validation

To validate the conceptualization, we present modeling diagrams to DE validation and refinement. Class and property names are validated with DEs to check their existence in the domain.

#### 3.2.3. Ontology encoding

After validation, OEs integrate the diagram in C3PO diagrams to generate a formal representation in OWL using Chowlk Converter.^6^ The class and property definition are written on the side in another tabular file so that they can be collaboratively edited by DEs and OEs. We generate the OWL file of the definition with OntoRefine, i.e., a tool to transform a tabular file into an RDF file using a template.^7^ We combine the Chowlk output file and definition file in Protégé (Musen, 2015) to consolidate and export a complete OWL file for C3PO module that we will use in our KG, exchanges with external parties, or publish in AgroPortal.

We supplement the C3PO KG with terms from controlled vocabularies used for property values. This “vocabulary part” of our KG contains information such as climate, unit of measure, and irrigation mode. To ease collaboration, it is maintained with VocBench (Stellato et al., 2015), an application that allows us to build ontology and thesaurus.

#### 3.2.4. Ontology evaluation

To evaluate the ontology module: (i) First, to validate the domain representation, we write SPARQL queries matching the competency questions and executed them on the ontology. The competency questions and the SPARQL queries are available.^8^ (ii) Second, to validate the structure and syntax, we use OOPS (Poveda-Villalón et al., 2014) to detect any classic ontology pitfalls. (ii) Third, to validate the embedded logic, we run the Pellet (Sirin et al., 2007) and Hermit (Glimm et al., 2014) reasoners to check the consistency of the ontology regarding the domain. (iv) Finally, to validate the usability, we explicitly used the ontology as a data model for an implemented application, which does run and fulfill its requirements (see Section 5 for details).

#### 3.2.5. FAIR ontology publication

The ontology publication phase consists of providing the OWL files and the documentation online according to FAIR principles. Regarding these principles, we published C3PO on GitLab in the form of a set of ontology module files, as presented in Section 4. We produced the documentation of each module using Widoco (Garijo, 2017), a tool that generates an HTML documentation from an OWL file. The publication phase also consists in adding metadata to the ontology to improve its description. We upload C3PO in AgroPortal and declare some metadata using the AgroPortal metadata schema named MOD (Dutta et al., 2015). To evaluate C3PO's fairness level (i.e., to which level our ontology adhere to the FAIR Principles), we used O'FAIRe (Amdouni et al., 2022), an ontology fairness evaluator for semantic resource proposed by AgroPortal. C3PO reaches 59% of fairness. More information about metadata standard is described in Section 4.2.

### 3.3. Knowledge graph construction

The knowledge graph constructed under C3PO consists of several heterogeneous data sources. The type of source impacted the way we imported data, as described hereafter.

#### 3.3.1. Domain expert data

As DEs are not Semantic Web experts, we support them in populating the C3PO KG with tabular files. Then, we use OntoRefine as was done when building the ontology to produce RDF files. The process is used to import characteristics of the cultivated plants and technical itineraries in the knowledge graph.

#### 3.3.2. Relational database import

Some agricultural databases that we wanted to be in C3PO KG were not available in RDF. We, thus, use tabular formats of the databases to which we apply a preprocessing, e.g., to produce URIs. In the tabular to RDF transformation process, value sets are encoded directly in our vocabulary that is built with SKOS (Miles and Bechhofer, 2009). Then, the files are transformed in OWL format with OntoRefine to produce an RDF dataset to be imported in the C3PO KG. We use this process for the integration of Basagri, a database containing information on agricultural chemical products and their uses with plants distributed by the Lexagri company.^9^ The database is updated daily, but it is not freely available. In section 4.1, we present the whole process and how we used the E-PHY ontology (Bouazzouni and Jonquet, 2021) to represent the Basagri chemical data.

#### 3.3.3. RDF linking

Semantic Web resources exist in agriculture, as presented in the related work. We manually linked our knowledge graph with other KGs such as TAXREF-LD and FCU to enhance the representation of plants with botanical and usage information (Darnala et al., 2022). The process involved DEs to produce and validate the set of links.

#### 3.3.4. Application data

As the ontology is designed to be used for crop planning, part of the data are from user input from applications (*Elzeard, La Serre des Savoirs* and *la Pépinière*).

The final C3PO KG is built by importing all the previously revised data in the same RDF database, i.e., GraphDB in our case.^10^ GraphDB was chosen to meet our requirements with respect to ease in dealing with RDF data directly, write and test SPARQL queries, create named graphs and reasoning features.

### 3.4. Ontology and knowledge graph maintenance

The ontology is updated each time a new use-case appears and requires an ontology development. Updates are also done to fix bugs remaining in the ontology or the knowledge graph. Moreover, as the ontology is published on GitLab, the submission of regarding bugs or improvements is possible. To update the knowledge graph, we implemented pipelines to produce RDF graphs from CSV files to enable continuous data development by DEs and improvement of the knowledge graph without an extensive need of an OE. To prevent direct insertion of triples in the knowledge graph and possible errors, we promote building of a new named graph for static data, i.e., in our case plant and input knowledge, each time a new batch of data is imported in the current knowledge graph. However, we could have problems of changing ids between two version of the knowledge graph, so a backup of each version of the knowledge graph is required.

Regarding user data present in the knowledge graph, we use Prov-O to track updates and provenance. Each update lists the modified instances, the user involved, and the time of the update. The update description is saved in JSON format as a value of a data property in the knowledge graph. Tracking updates allows us to know who performs the update and recover from previous timestamps if needed.

## 4. The C3PO ontology and knowledge graph

In this section, we describe the current version of the C3PO and specific development strategies for both the ontology source file and the knowledge graph. We divided the ontology in several modules, each representing a specific sub-domain of interest for vegetable agriculture planning. We chose such modular representation as we identified/extracted from the conceptualization step several sub-domains, which could work independently but still strongly related. Furthermore, modules helped during the conceptualization to divide the work and focus on sub-domains instead of the whole area. As an example, we divided the representation of plant knowledge and plot organization into two distinct modules. We divided the modules between support modules and domain modules. Support modules are used in almost all the domain modules to improve reusability between the modules. Domain modules are representing sub-domains of the C3PO domain. Table 2 presents the module, their namespace, and the color used in the Figures of Section 4. We published the competency questions for Plant, CropManagment and Plot module on the documentation, and the SPARQL queries.^11^^,^^12^

**Table 2:** C3PO modules and their URIs and namespaces.

**Module name**	**Module URI**	**Module namespace**
**Support modules**		
Time	http://www.elzeard.co/ontologies/c3po/time	c3potime
Vocabulary	http://www.elzeard.co/ontologies/c3po/vocabulary	c3povocab
Parameter	http://www.elzeard.co/ontologies/c3po/parameter	c3poparam
**Domain modules**		
Plant	http://www.elzeard.co/ontologies/c3po/plant	c3poplant
Plot	http://www.elzeard.co/ontologies/c3po/plot	c3poplot
Crop Management	http://www.elzeard.co/ontologies/c3po/cropManagement	c3pocm
Admin	http://www.elzeard.co/ontologies/c3po/admin	c3poadmin
Supply	http://www.elzeard.co/ontologies/c3po/supply	c3posupply
Sale	http://www.elzeard.co/ontologies/c3po/sale	c3posale

### 4.1. Ontology modules and knowledge graph

#### 4.1.1. Support modules

**Time module**

**Figure 3:**
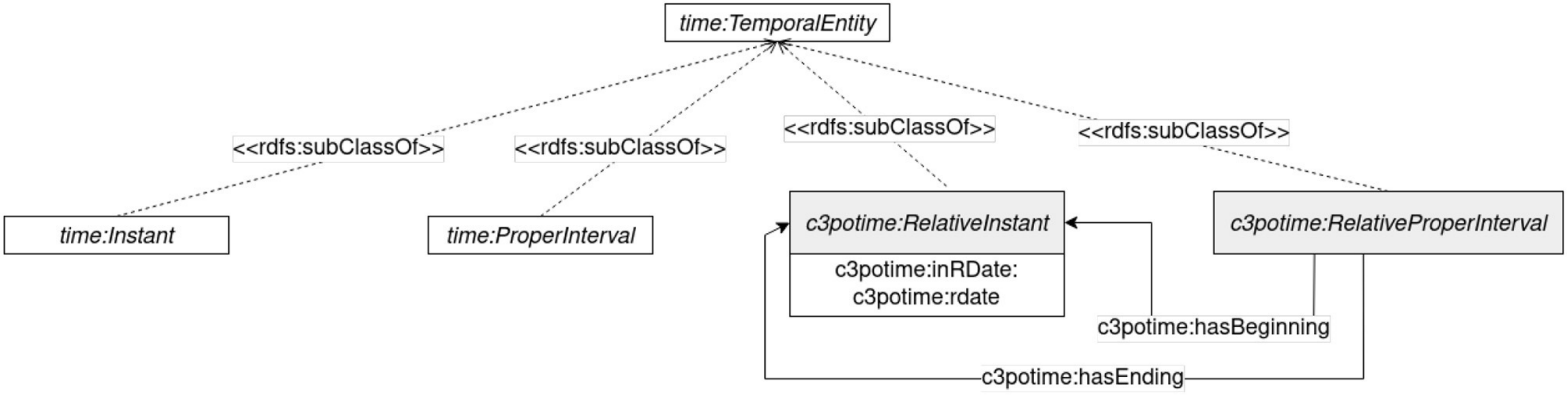
Time module representation.

The Time module extends the Time Ontology to be able to represent c3potime:RelativePropertInterval composed of time:RelativeInstant, as shown in Figure 3, while the respective representations of time instants are not placed in a specific year. This is important for representing cultivation dates for any crop that might occur in a different year. An example of a c3potime:RelativePropertInterval could be the date interval between two time:RelativeInstant: the 16th of September and the following 2nd of February. This allows to create patterns reusable every year. Moreover, it allows sharing of information that could be reused at any time without reference of the year. time:RelativeInstant has the data property c3potime:inRDate with a type c3potime:rdate formating “_Y_M_F_W_D” where “_” represent a number, “Y” a year, “M” a month, “F” a fortnight, “W” a week, and “D” a day. The previous example should be represented as “0Y9M16D” for the 16th of September and "1Y2M2D" for the 2nd of February.

**Vocabulary module**

The Vocabulary module corresponds to several closed lists of qualitative values represented as SKOS thesaurus. The SKOS thesaurus has several top concepts dedicated to a specific list: units, climate characteristics, culture modes, etc. Any skos:Concept instances are linked to instances from other C3PO modules using specific object properties. For example, the instance of the class c3poparam:Parameter is linked to any narrower concepts of c3povoc:Unit using the property c3poparam:hasParameterUnit. Those skos:Concept instances representing unit are partially aligned with QUDT (Hodgson et al., 2014) instances, i.e., a knowledge graph representing the various standard quantity kind and unit.

**Parameter module**

The Parameter module corresponds to a representation of numeric parameters such as weight and volume. The module is composed of a class c3poparam:Parameter with a measured, minimun, and maximum value. The class is specialized for each measurement type. Each parameter class have a constraint to specify its unit, defined in the Vocabulary module.

For example, the class c3poparam:Yield will store all the yield measurement with the associated unit c3povoc:YieldUnit and is defined as follows:


c3poparam:Yield rdf:type owl:Class ;
   rdfs:subClassOf c3poparam:Parameter ,
      [ rdf:type owl:Restriction ;
         owl:onProperty c3poparam:hasParameterUnit ;
         owl:allValuesFrom [owl:intersection
(skos:Concept
            [ rdf:type owl:Restriction ;
            owl:onProperty skos:broader ;
            owl:hasValue c3povoc:YieldUnit]) ;
      rdf:type owl:Class]
] .


#### 4.1.2. Domain modules

**Plant module**

**Figure 4:**
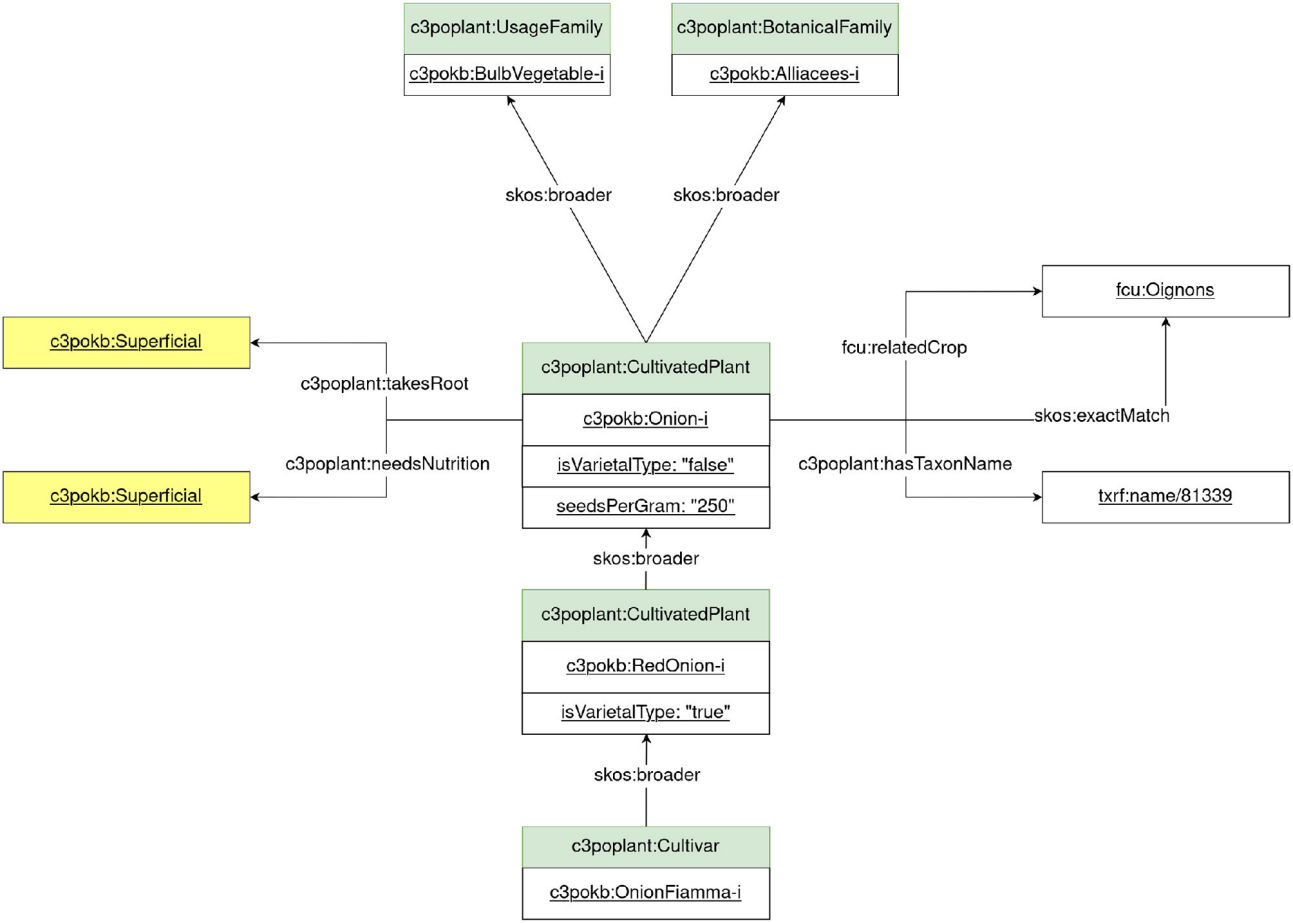
Plant module representation.

The Plant module represents cultivated plant taxonomy from a farmers' viewpoints. Plants are described by the class c3poplant:CultivatedPlant with characteristics such as crop seasons, watering needs, or nutritional requirements. Plants are hierarchically organized under a taxonomy representing as a SKOS thesaurus. This taxonomy has different levels as follows: plant family, cultivated plant, varietal type, and cultivar, as shown in Figure 4. Varietal type is an intermediate level between cultivated plant and cultivar that represents some physical characteristics of crops that farmers refer to while defining the market outlets. This level does not belong to a botanical scientific taxonomy. Again using the onion example of the previous section, the botanical and usage families of the onion are, respectively, Amaryllidaceae and bulb vegetables. We defined several varietal types such as yellow onions and red onions. Fiamma is an example of a red onion cultivar. The module also represents crop succession and association information. Figure 4 presents the main classes of the Plant module instantiated with the onion example. A Plant knowledge graph was built under the Plant module with the help of agronomists and is linked with FCU and TAXREF-LD. In future, the module will be extended to improve the representation of trees, which will be useful with regard to fruit crops.

**Plot module**

**Figure 5:**
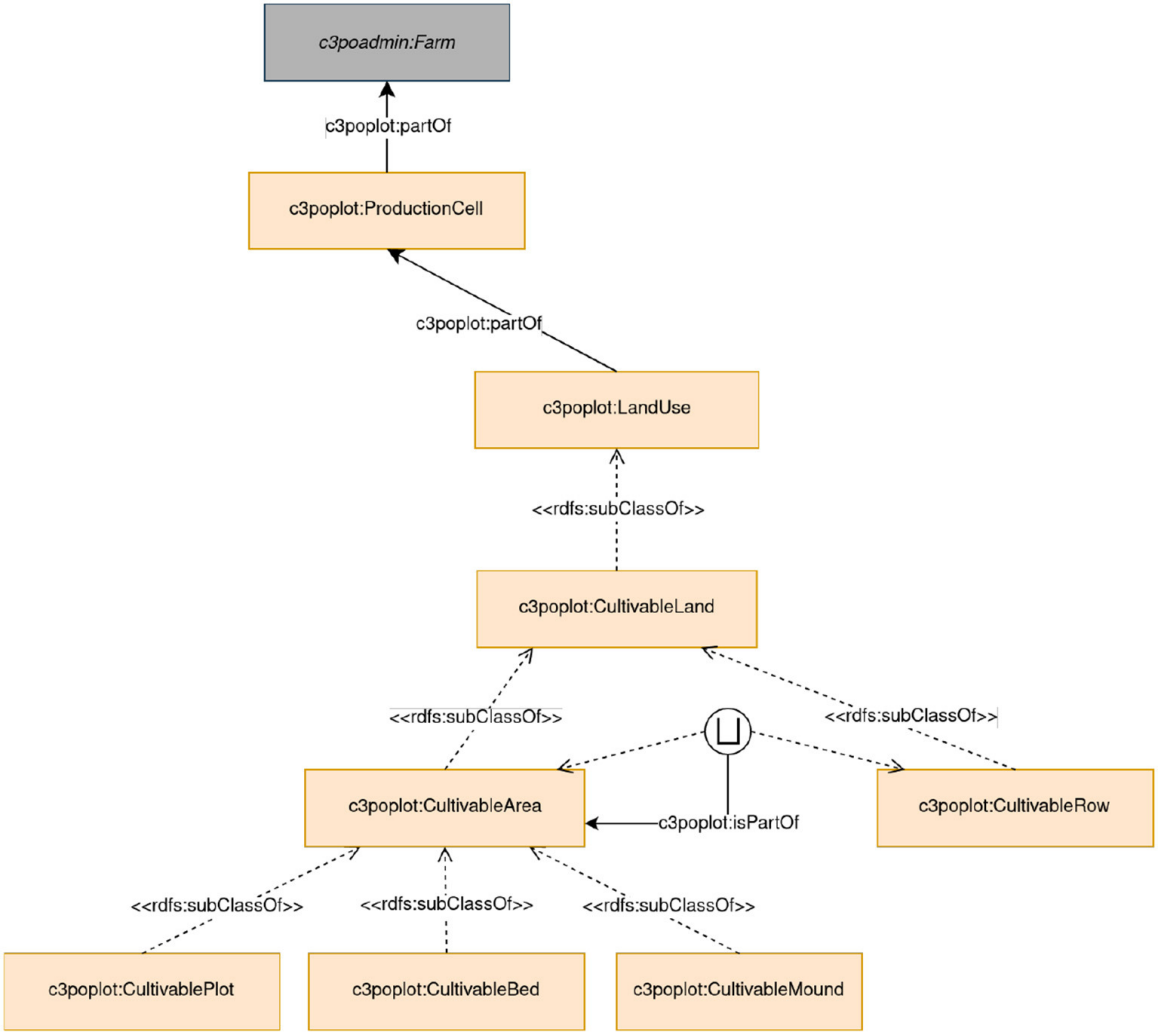
Plot module representation.

The Plot module represents the spatial organization of plots on the farms. It contains the representation of a c3poplot:ProductionCell, which is an occupation of legally registered land with an address and an id. In addition, the farmer-driven spatial organization is represented with the possibility of creating c3poplot:CultivablePlot and c3poplot:CultivableBed within the plots, which may be useful for diversified vegetable crop farmers growing multiple crops on the same plot or the same row. Irrigation systems and landscape elements such as meadows present on farms are also represented. Figure 5 presents the main Plot module classes.

In future, the module will be extended to improve the representation of irrigation systems and landscape elements by taking into account all the specific features of the lands, especially in areas of ecological interest that are required to the farm to get different certifications.

**CropManagement module**

**Figure 6:**
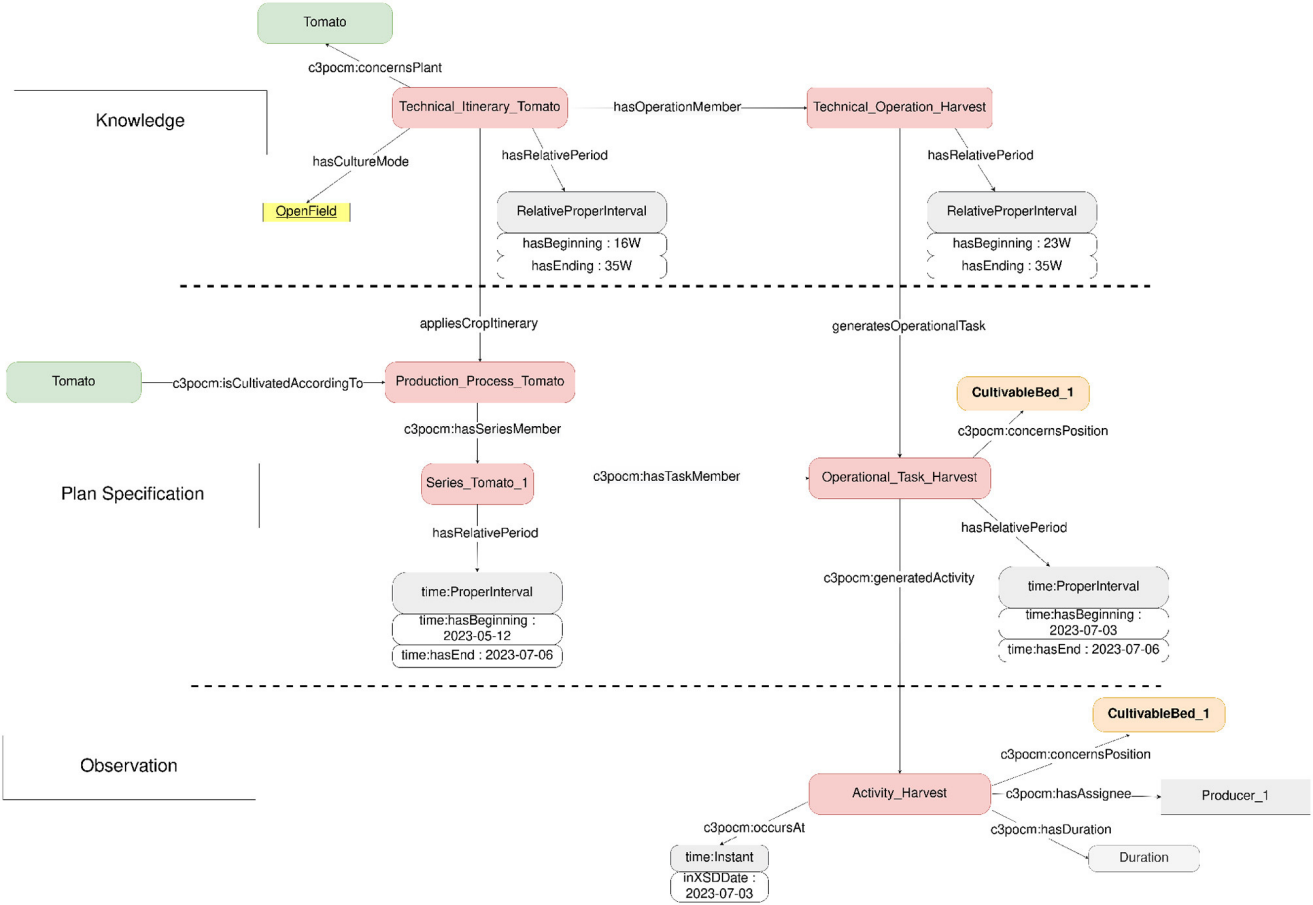
CropManagement module representation.

The CropManagement module represents technical itineraries and farming processes for task planning and recording. As noted previously, we studied the three ValueFlows representation layers: plan specification, plan and plan execution. Plan specification is built by instantiating the c3pocm:CropItinerary class, representing generic technical itineraries created by farmers and agronomic experts. A c3pocm:CropItinerary consist of a set of tasks. One generic task is represented as a c3pocm:TechnicalOperation. These generic technical itineraries are linked to a c3poplant:CultivatedPlant from the plant module and have parameters such as season and soil type. c3pocm:TechnicalOperation are farming processes such as planting or harvesting, which are described with c3potime:RelativeProperInterval to give a time range where the task could be applied. Plan is built by instantiating the c3pocm:ProductionProcess class for each crop. c3pocm:ProductionProcess is composed of a set of c3pocm:OperationalTask planned in the farmer's calendar. Farmers often rely on series principles, i.e., they grow the same type of crops with (more or less) the same set of tasks, but on a different plot and at a different time to achieve a continuous flow of crop production. As shown in Figure 6, we integrate the c3pocm:Series classes to represent this principle. Plan execution is built by instantiating the c3pocm:Activity class. An c3pocm:Activity represents one task carried out. c3pocm:OperationalTask and c3pocm:Activity are tasks that happened on crops and land, so we add a property named c3pocm:concernsPosition to link a task and the instance of the c3poplot:LandUse class from Plot module. As an example to present the difference between c3pocm:OperationalTask and c3pocm:Activity, a possible instanciation of c3pocm:OperationalTask could be a harvest happened between 4 July 2022 and 6 July 2022, with a certain estimated yield. During the execution, an instantiation of c3pocm:Activity is made for each day (4 July 2022, 5 July 2022, and 6 July 2022), with the real yield obtained per day. The three types of task c3pocm:TechnicalOperation, c3pocm:OperationalTask and c3pocm:Activity are linked to instance of c3pocm:FarmingPractice. c3pocm:FarmingPractice is specialized in many sub-classes, representing various farming tasks such as harvesting or planting. Each sub-class has its own parameters. The three layers help farmers to analyze their production and decide what should be changed the following year to improve their productivity. Figure 6 presents the main classes of the CropManagement module.

The CropManagement knowledge graph built under the C3PO CropManagement module has been partially populated with the help of agronomists, especially regarding the integration of instances of c3pocm:CropItinerary.

In future, the module will be extended to link the harvested crops with the Sales module.

**Admin module**

The Admin module represents agents and organizations and their administrative information as users of the applications. It relies on existing standard ontology modules or vocabularies, such as FOAF (Graves et al., 2007) for the representation of people and organizations and Event Ontology (Raimond and Abdallah, 2007) for events. We created subclasses from these resources to integrate c3poadmin:Farm, c3poadmin:Producer and c3poadmin:Cooperative representations.

**Supply module**

**Figure 7:**
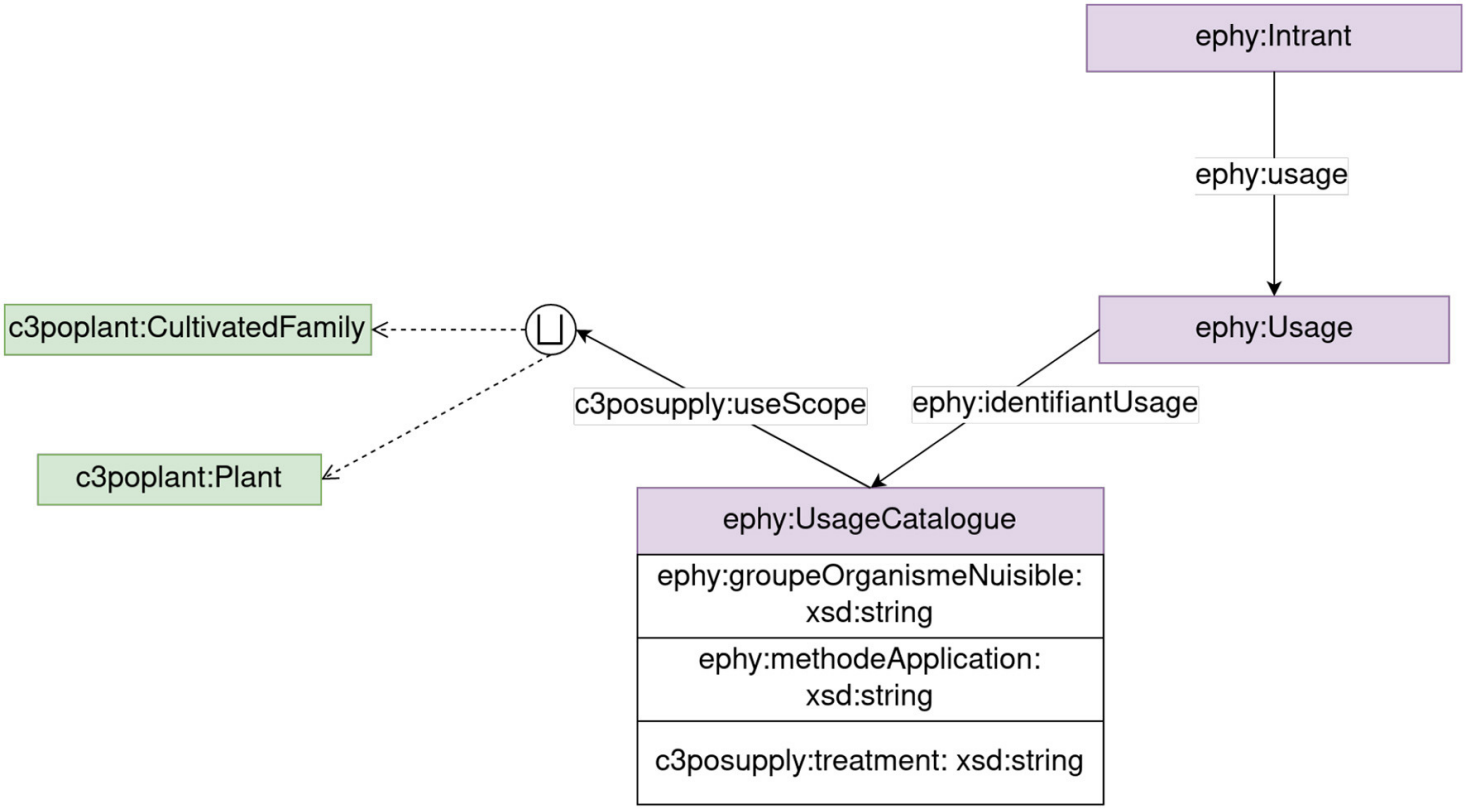
Supply module representation.

The Supply module represents agricultural input and equipment that farmers could use. The module allows users to represent input as ephy:Intrant and their usages as ephy:Usage. A ephy:Usage is the combination of a product, plant, or family, a targeted pest and the application method of the product. This combination defines properties such as the maximum dosage allowed or the number of possible applications. Figure 7 presents the main classes of the CropManagement module. The Supply module currently extends the E-PHY ontology to integrate Basagri data. The E-PHY ontology is an ontology produced to represent the French E-Phy catalog of plant protection products. Basagri is a private dataset containing information regarding regulatory data on agricultural inputs in France. The dataset is proposed as a set of files in CSV format. We implemented a pipeline to transform the CSV format into an RDF knowledge graph under the E-PHY ontology as it fulfilled our requirements regarding agricultural inputs. We extracted different information such as the dosage authorized for a product regarding a plant or the number of days required before the farmers return to the plot or harvest, from the Basagri files. We, then, built URIs for input using their marketing authorization (AMM, a code delivered France for authorized chemical products) as in the E-PHY proposition. We created vocabularies using SKOS thesaurus representations for closed lists such as the type of product function (insecticide, herbicide, etc.). We also compared the list of crops of Basagri and C3PO to find similarities based on labels and connect ephy:Usage to instance of c3poplant:CultivatedPlant or c3poplant:CultivatedFamily from the plant module. We used the Levenstein distance (Levenshtein et al., 1966) to deal with slight differences such as singular/plural names. In future, the module will be extended to improve the representation of farming equipment. Moreover, the alignment of Basagri and C3PO crops will also be improved to enhance the number of similarities between the two databases.

**Sale module**

The Sale module organizes the stocks and the product delivery. The module is still under construction and will be updated and combined with the DataFoodConsortium ontology which represents the supply chain and delivery process in the food distribution system.^13^

### 4.2. Statistics and availability

**Figure 8:**
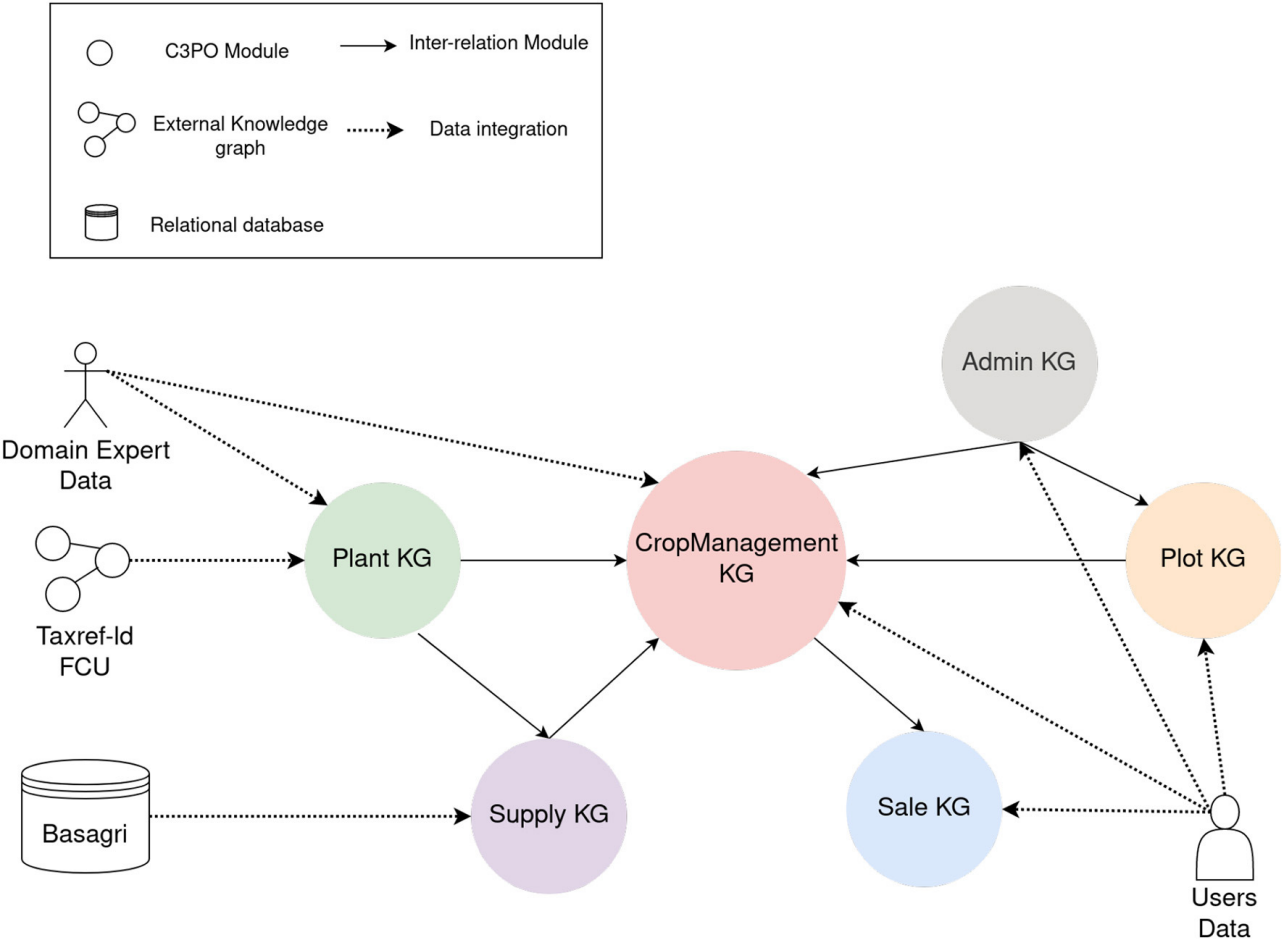
Components of C3PO's knowledge graph.

The C3PO knowledge graph currently consists of 4,647 axioms, 236 classes, 211 object properties, 71 data properties, and 270 individuals mostly contained in the Vocabulary module. Moreover, the KG is currently composed of 8,402,495 triples, of which 3,025,790 are explicit and 5,376,790 are implicit. All of the ontologies are available under the Creative Commons Attribution 4.0 International license (CC-BY 4.0). Figure 8 presents different components of C3PO's knowledge graph (module, inter-module relations, and data source).

A sub-part of the knowledge graph containing the Plant module and the CropManagement module is available on GitLab under an Attribution-ShareAlike 4.0 International license (CC BY-SA 4.0). The other parts are also not public because they concern users' data or are licensed, e.g., Basagri.

According to the MIRO guidelines (Matentzoglu et al., 2018), hereafter in Table 3, we present the current version (1.0) of C3PO. Only the MIRO “basic” guidelines are reported here, but we have incorporated as much metadata as possible in the C3PO OWL source file, according to the MOD specifications (Dutta et al., 2015).

**Table 3:** C3PO information following basics MIRO guidelines.

**Basics MIRO guidelines**	**C3PO information**
Admin	http://www.elzeard.co/ontologies/c3po/admin
A.1 Ontology name	Crop planning and production process ontology
A.2 Ontology owner	•Baptiste Darnala (Elzeard) •Florence Amardeilh (Elzeard)
A.3 Ontology license	Attribution-ShareAlike 4.0 International (CC BY-SA 4.0)
A.4 Ontology URL	http://www.elzeard.co/ontologies/c3po
A.5 Ontology repository	https://gitlab.com/serre-des-savoirs/c3po https://agroportal.lirmm.fr/ontologies/C3PO

C3PO and its modules are uploaded on AgroPortal. We published each module as a view of the ontology in AgroPortal project. We edited some metadata on the global level: C3PO. We improve the FAIR score of the ontology by following the AgroPortal guidelines during the Metadata AgroHackathon in August 2022.^14^

## 5. The ontology and knowledge graph in use

We are developing the ontology and knowledge graphs in the context of multiple application development for knowledge sharing and crop planning. These applications are built by Elzeard and developed in the framework of the MESCLUN DURAB, PACON (Morel et al., 2023), and D2KAB (Aubin et al., 2019) research projects.^15^ These applications help to assess the ontology consistency regarding the domain and the quality of the data integrated in the knowledge graph.

### 5.1. *"Serre des Savoirs"*

The so called “*Serre des Savoirs*” web portal is under construction to access open data in the C3PO KG related to cultivated plants and technical itineraries. The knowledge graph content is described with the Plant and CropManagement modules. For the CropManagement module, the application will query only the CropItinerary and TechnicalOperation instances. The web portal will directly query the knowledge graph and make it accessible for non-Semantic Web experts such as farmers and agronomists. The development of the application has impacted the development of the Plant and CropManagement module and the needs in terms of information required in the application.

**Figure 9:**
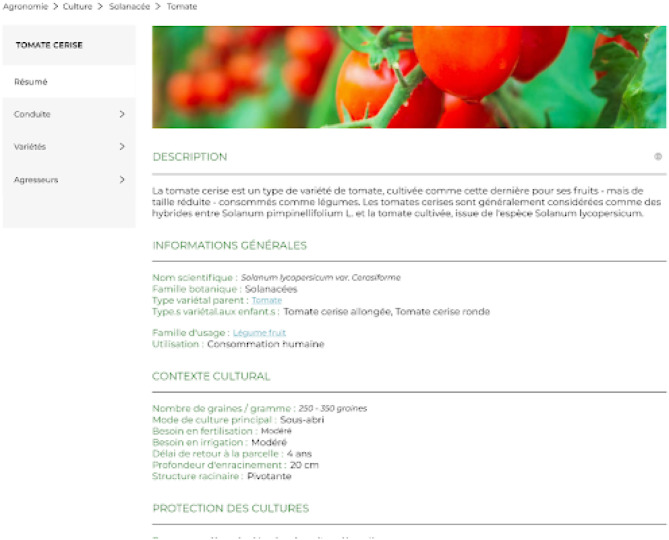
Descriptive page of the tomato in *La Serre des Savoirs*.

A screenshot of the descriptive page of the tomato is presented in Figure 9. The information displayed provides a general description of this vegetable including the species scientific name, the cultivating families, and varietal types described in Section 3.2. Moreover, information on the cultivation context is provided, such as the irrigation and nutrition needs of the plant. Several competency questions of plant module are used to build this page presented as follows: ^16^

1. What is the plant's botanical species?
2. What is the plant's botanical family?
3. What is the scientific name of the botanical taxon (species or family)?
4. What is the plant's usage family?
5. What are the varietal types of a plant?
6. How much does the seed of a plant weigh (seeds per gram)?
7. How much water does a plant need?
8. How deep does a plant's root system go?
9. How much nutrients does a plant need?
10. How long does it take for a plant to return to the plot?

To illustrate the querying of the C3PO KG, the SPARQL queries corresponding to CQ2 and CQ10 are presented as follows:


PREFIX c3poplant: <http://www.elzeard.co/ontologies/c3po/plant#>
PREFIX skos: < http://www.w3.org/2004/02/skos/core#>



select ?CultivatedPlant ?BotanicalFamily where {
   ?CultivatedPlant a c3poplant:CultivatedPlant .
   ?BotanicalFamily a c3poplant:BotanicalFamily .
   ?CultivatedPlant skos:broader+
?BotanicalFamily . }



PREFIX c3poplant: < http://www.elzeard.co/ontologies/c3po/plant#>
PREFIX c3poparam: < http://www.elzeard.co/ontologies/c3po/parameter#>



select ?CultivatedPlant ?PlotReturnTime
?parameterValue ?minValue ?maxValue
?ParameterUnit where {
    ?CultivatedPlant c3poplant:hasPlotReturnTime
?PlotReturnTime .
    Optional {?PlotReturnTime
c3poparam:parameterValue ?parameterValue .}
    Optional {?PlotReturnTime c3poparam:minValue
?minValue .}
    Optional {?PlotReturnTime c3poparam:maxValue
?maxValue .}
    ?PlotReturnTime c3poparam:hasParameterUnit
?ParameterUnit . }


### 5.2. Decision support applications

**Figure 10:**
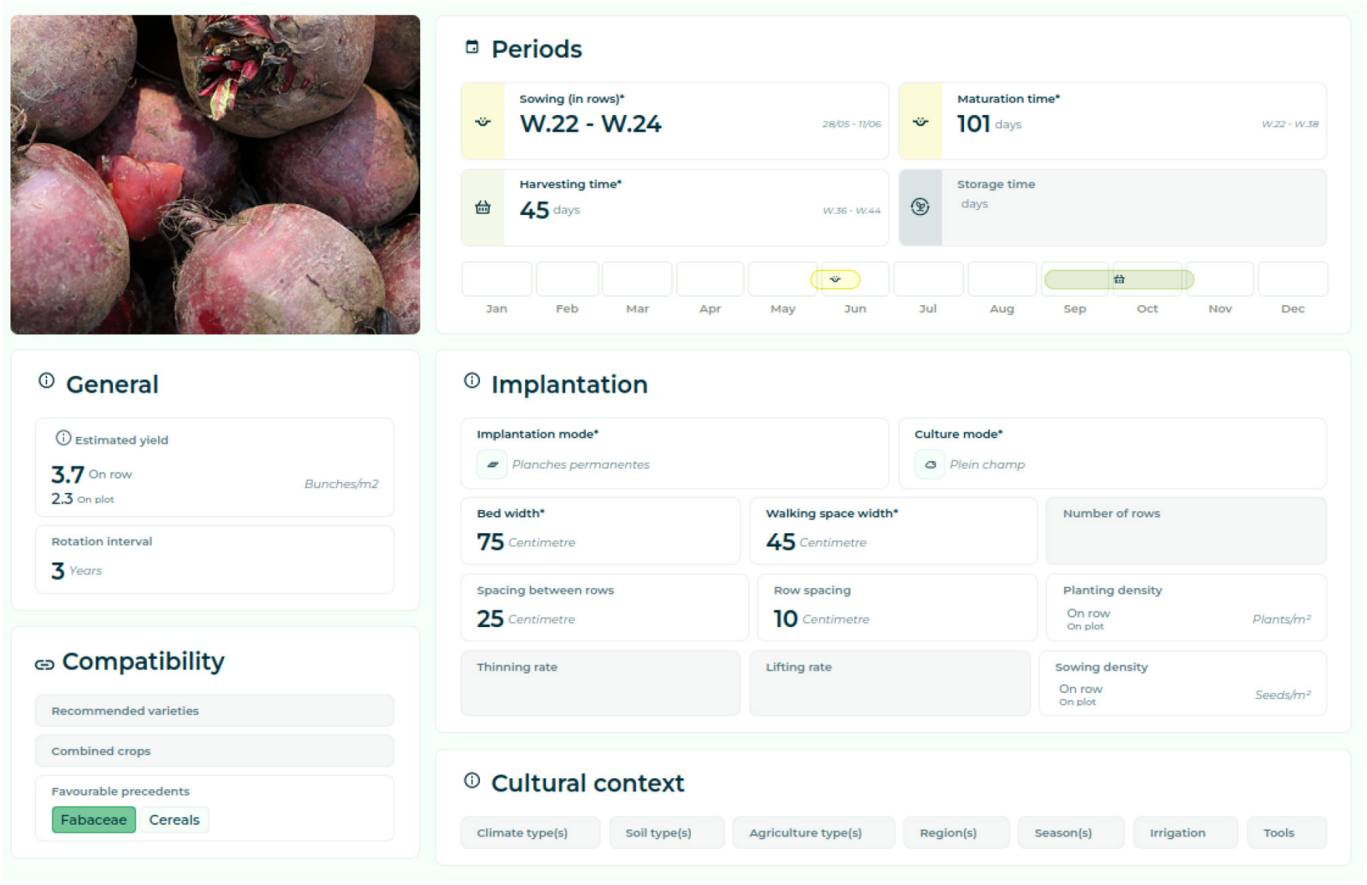
Technical itinerary of the beet in *Elzeard*.

In addition, the C3PO KG was used to build two applications for farmers: *Elzeard* and *La Pépinière*. *Elzeard* is a web application where farmers describe their farms with their locations and plot organization. The farmers can, then, build the plan for their crops and related tasks, organize their farm workers' schedules, and choose the inputs to use. C3PO is used as the data model for the web application. The knowledge graph built with plant, technical itineraries, and input knowledge is queried to help farmers access decision-support information. C3PO is used in *Elzeard*. Figure 10 presents a screenshot of a technical itinerary in *Elzeard*. The list of competency questions involved in building this webpage come from the CropManamgement module:^17^

1. How long does it take for a plant to emerge in this technical itinerary?
2. How long does a plant grow in this technical itinerary?
3. How long does it take to harvest a plant in this technical itinerary?
4. What is the shelf life of a plant in this technical itinerary?
5. What is the estimated overall workload for this technical itinerary?
6. What are the tasks involved in this technical itinerary?
7. When is the best time to plant in this technical itinerary?
8. Over what period will I be able to spread out the harvests for this technical itinerary?
9. Which task must be carried out before or after another task in this technical itinerary?
10. What is the forecast yield for this technical itinerary?
11. Which varieties are recommended for this technical itinerary?
12. What is the expected yield for this technical itinerary?

To illustrate the querying of the C3PO KG, the SPARQL query corresponding to CQ7 is presented as follows:


PREFIX c3pocm: < http://www.elzeard.co/ontologies/c3po/cropManagement#>
PREFIX c3potime: < http://www.elzeard.co/ontologies/c3po/time#>
select ?CropItinerary ?FarmingPractice
?beginningDate ?endingDate
where {
    ?CropItinerary a c3pocm:CropItinerary .
    ?CropItinerary c3pocm:hasOperationMember
?Operation .
?Operation c3pocm:implements ?FarmingPractice .
?FarmingPractice a c3pocm:PlantingProcess.



Optional {
    ?Operation c3pocm:hasRelativePeriod ?Period .
    ?Period c3potime:hasBeginning ?Beginning .
    ?Beginning c3potime:inRDate ?beginningDate .



    ?Period c3potime:hasEnding ?Ending .
   ?Ending c3potime:inRDate ?endingDate .
  }
}


**Figure 11:**
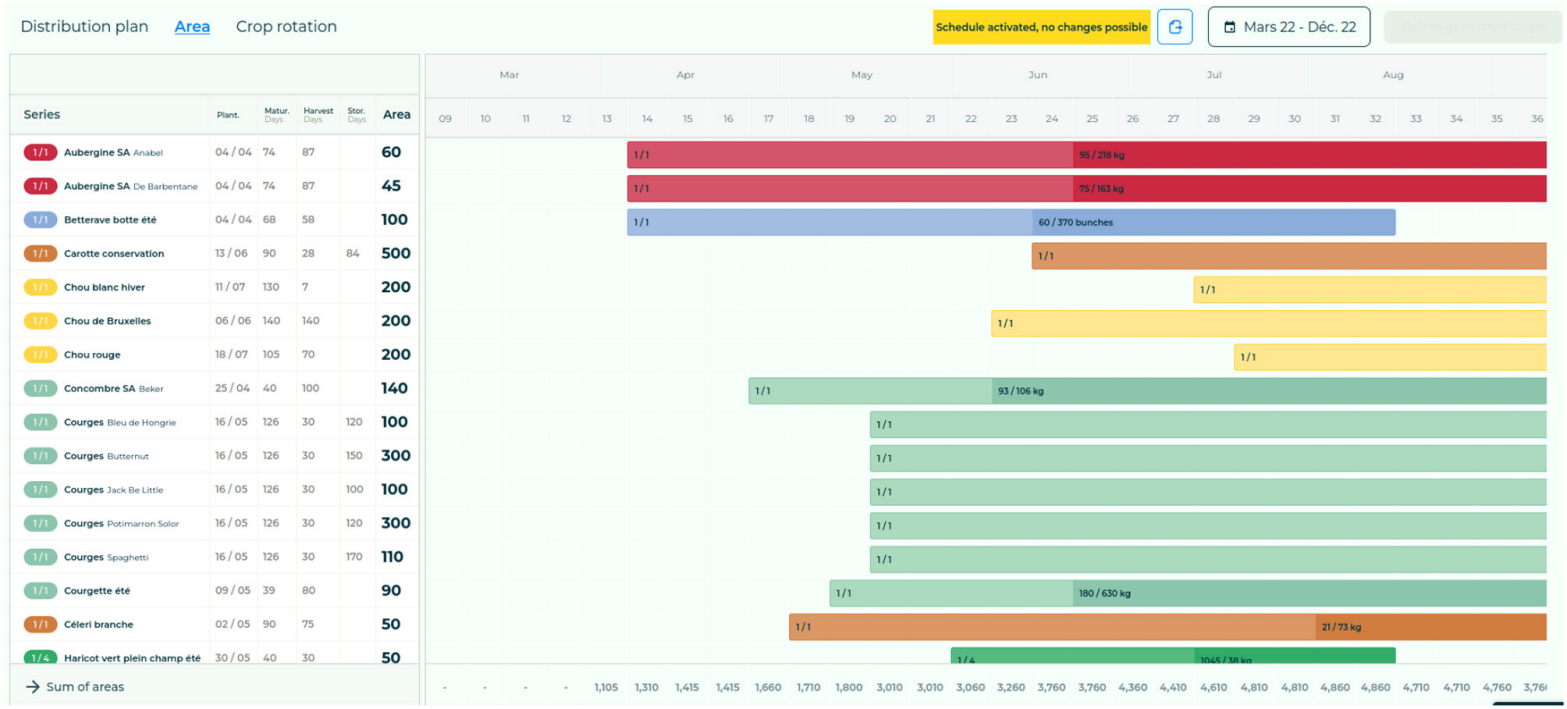
Crop planning of a farmer in *Elzeard*.

Figure 11 presents a screenshot of a planning made by a farmer, both made in the application *Elzeard*. Figure 10 is composed of several pieces of information such as the cropping period and the cultivating tasks. Figure 11 gives an overview of all the crops of a farmer in a period and the commercial needs in terms of harvested crop. The list of competency questions involved in building this webpage come from the CropManagement module:^18^

1. How many series have I planned for this crop?
2. What is the period of each series that I have planned for this crop?
3. Which variety is associated with this series?
4. What is the surface area associated with this series?
5. What are the planting distances between my seedlings or plants for this series?

**Figure 12:**
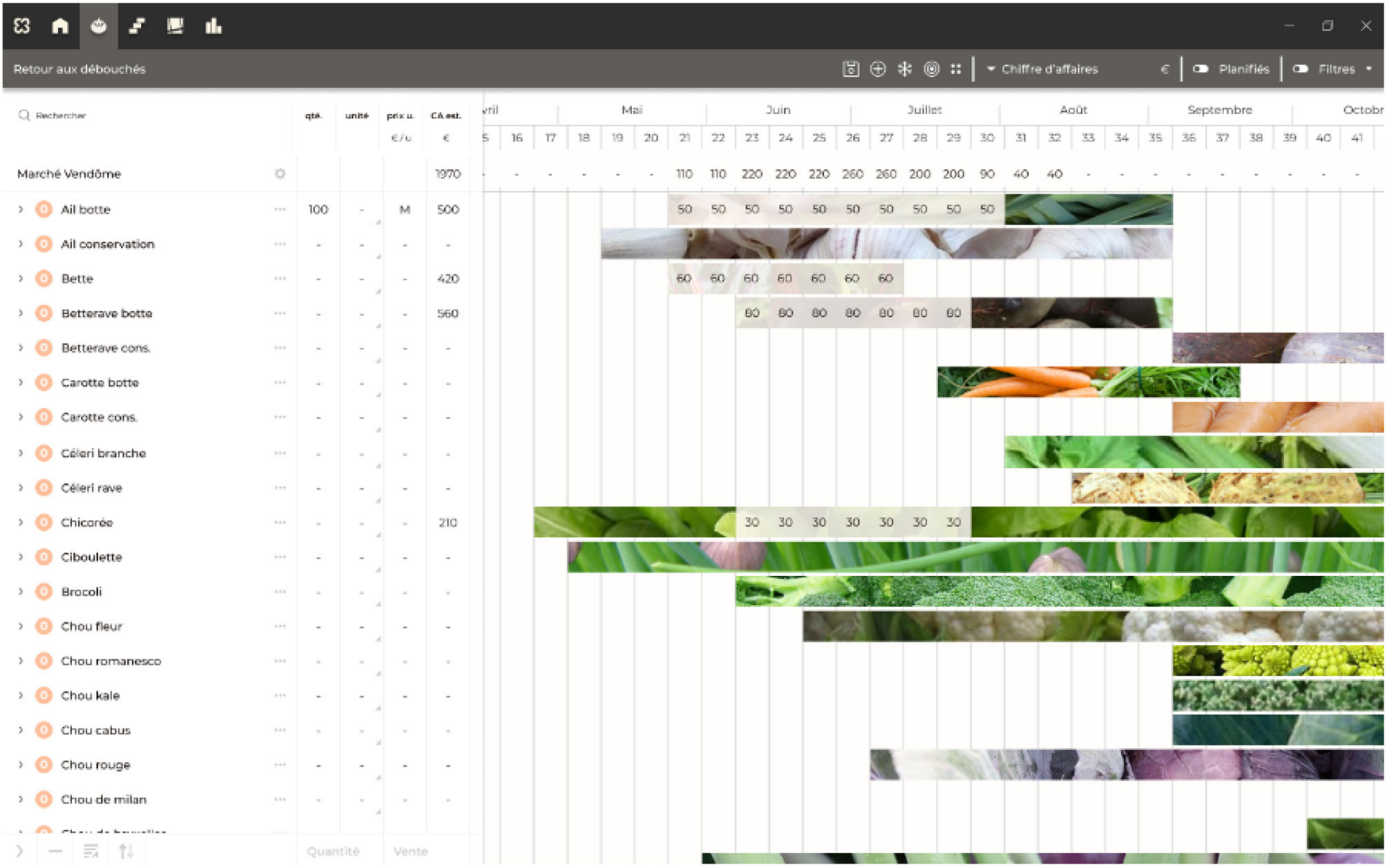
Crop planning of a farmer in *La Pépinière*.

*La Pépinière* is an application under development to help beginner farmers design their farms and future productions. The application has an educative feature, whereas *Elzeard* is production-oriented purpose. *La Pépinière* uses the Plant, CropManagement, and Plot modules as data models and has access to the same knowledge present in *La Serre des Savoirs* to help farmers. Figure 12 presents a screenshot of the *La Pépinière* application. The competency questions used are the same as presented in Figure 11.

## 6. Discussion and difficulties

We encountered several difficulties during C3PO development process. Concerning C3PO development, the diversified vegetable agricultural domains and the multiple viewpoints were complex to represent in a single ontology. The division into modules eases the development by allowing to work in multiple subdomains independently. However, naming problems of classes shared between multiple modules could arise and should be checked when updating the ontology. In addition, this complexity required the involvement of multiple domain experts such as farmers, agronomists, taxonomists, and retailers to have a better vision of the domain and the needs the ontology should meet.

As illustrated, C3PO, even if not yet perfect, was developed primarily to serve multiple applications, and thus, a usable ontology had to be produced quickly. The Agile development methodology, which eases the release of several iterative ontology versions already usable by application developers, was suitable. However, new domain discoveries could impact previous development choices and lead to refinement of the ontology impacting the knowledge graph structure. Moreover, our methodology helps us to keep track all steps of development process. The files (Chowlk schema, text description,...) produced during the specification and conceptualization steps are used to document C3PO.

Regarding cooperation with the development team and domain experts, we had to use tools that are easily understandable by non-Semantic Web users. The graphic notation proposed by Chowlk helped to produce schemes that would be understandable by different actors and directly convertible into OWL format, but this generated a more complex ontology engineering workflow, otherwise we would have simply used Protégé in group.

In the documentation writing process, we included definitions of classes and properties in shared documents to improve collaboration between DEs and OEs. It was important to provide understandable detailed definitions to enhance the model reusability.

During the C3PO conceptualization process, various difficulties were encountered according to the concerned module. For the Plant module, our aim was to create a module that represents a plant taxonomy with multiple viewpoints (botanical and agricultural), to have agricultural information on plants and ease the link with different heterogeneous knowledge graphs. This led to the typing of instances as skos:Concept and owl:Class. SKOS offers the possibility of creating the plant organization and links with other skos:Concept, while OWL helps to create classes and properties. For the Supply module, the transformation of an existing ontology (E-PHY) led to checking if the ontology was usable and the changes required to meet our needs. The most important changes were made when we opted to change the domain or the range of a property. In that case, we chose to create a new property. The main drawback of this method is the E-PHY ontology, and its properties are provided in French while C3PO is built in English, so the URIs of the module are not in the same languages. Linguistic uniformization could be applied in future.

During the C3PO KG building process, multiple and heterogeneous sources of data required us to build multiple data integration pipelines. For the Plant module, we had to test different data integration scenarios with domain experts. As previously noted, we ended by using tabular files. This method eases the integration of domain expert data but could lead to problems. As multiple spreadsheets are used, problems of misspelled URIs can occur and lead to missing connections in the graph. We overcame this problem by creating a list of SPARQL queries to check the consistency of the graph, find errors, and fix them before importing the data in the graph. We also had difficulty connecting our instances with TAXREF-LD as taxons are represented with owl:Class, and scientific names are represented by instances of skos:Concept. We decide to link our C3PO instances with skos:Concept using specific C3PO's properties. Notably, a link between a class and an instance is possible only with the property rdf:type. Regarding the data imported from the applications, problems were encountered due to wrong data integration or failed knowledge graph updates. For instance, properties were duplicated instead of being renamed after an ontology update. Here, we used SPARQL queries to address these issues. We recommend to update the knowledge graph by exporting the data, applying the change, and importing in a new knowledge graph. Thus, we keep track of changes to be able to roll back. In this way, the knowledge graph is not updated directly.

About the reasoning aspect, we define some constraints on C3PO's classes to check the quality of users' input data (c3poparam:Parameter). However, we do not check the expert data extracted from reference sources (TAXREF-LD, FCU, etc.). Unfortunately, mistakes may happen on this source that will cause inconsistencies. Thus, we should apply reasoning and other checking processes on those part of the graph in future.

The knowledge graph is not fully opened. A subpart regarding information about plants and technical itineraries is accessible in GitHub and through a SPARQL endpoint. User information saved in the Plot, Admin and CropManagement modules remain private. Sharable information will be accessible through the “La Serre des Savoirs” Web Portal. This portal will be enriched with data already aggregated, and users will have the possibility to enrich “La Serre des Savoirs” directly.

Regarding the interoperability and reusability challenges, we applied different workflows. First, we linked a subpart of our plant instances with other knowledge graphs (FCU and TAXREF-LD). However, we recreate the concepts, we aggregate a subpart of the knowledge such as labels, and we keep the alignment. We process as well to control the management of the terminology and to enrich it with multiple sources. In addition, we are using core domains ontologies such as FOAF, Prov-O, or Time ontology. Regarding future development to reuse or align with domain ontologies, we prefer to align our concepts instead of import external concepts. This choice is made regarding the context of industrial development, as we cannot ensure that external ontologies will be sustainable. Align instead of import offer the possibility to keep the control on C3PO. In addition to that, major concepts such as technical itineraries do not exist in other ontologies, which reduce the possibility of reusing this part of the graph. Finally, domain ontologies reused in C3PO are stored in AgroPortal repository and were found through this repository. Thus, ontologies not declared in the repository were not studied during our conceptualization step. However, we may miss some interesting ontologies such as PestOn (Medici et al., 2022), which means that we should update regularly our state-of-the-art research.

## 7. Conclusion and future work

Diversified vegetable farming is complex, and many parameters have to be taken into account for decision support. We built the Crop Planning and Production Ontology (C3PO) and its Knowledge Graph to help farmers in their choices. The ontology is divided into several modules to represent a specific part of the domain. The knowledge graph is created from heterogeneous data sources (other knowledge graphs, relational databases, or user/expert data). The C3PO KG is the backbone of three web applications and aims to give farmers access to information to support their planning and monitoring decisions. The open part of the knowledge graph brings novel aspect as no representation of technical itineraries exists for vegetables farmers. This knowledge has not been formalized yet and serve as a basis for reusability of common technical itineraries shared in different sources. Future studies will be continued to the referencing and sharing of technical itineraries to create a collaborative knowledge base through our web portal “La Serre des Savoirs” currently in development.

These applications–not all yet in production–already pre-validate C3PO as an “application ontology”, but future reuses will also validate C3PO as a “domain ontology”. The methodology presented in this study is based on LOT and SAMOD methodologies, and we highlighted how we implemented each process in an application development operation. Various domain expert partners were included in our approach to assess and identify the main concepts and properties: scientists (from the D2KAB, MESCLUN DURAB research project) and agricultural professionals (crop farmers, networks of agricultural advisors, and teachers). C3PO is available on GitLab as an open source project that can be reused and contributed to and published in AgroPortal to facilitate its discovery and reuse. In future studies, we will extend and improve the ontology to include equipment, farm components (e.g., irrigation structures or meadows currently present in the ontology but need refinement), and pests and diseases. We will also improve the ability of the ontology to make inferences on the data based on agricultural knowledge. We will improve the interoperability of C3PO and create alignment with other semantic resources. Finally, we will extend the scope of the ontology and knowledge graph in order to be able to model other types of crop production, such as arboriculture or agroforestry.

## Data availability statement

TAXREF-LD: The version 15.2 of TAXREF-LD graph used for this study can be found in the AgroPortal repository https://agroportal.lirmm.fr/ontologies/TAXREF-LD. The github repository is https://github.com/frmichel/taxref-ld. The SPARQL EndPoint is https://taxref.mnhn.fr/sparql. FCU: The version 3.3 of the FCU thesaurus used for this study can be found in the AgroPortal repository: https://agroportal.lirmm.fr/ontologies/CROPUSAGE. The gitlab repository is https://gitlab.irstea.fr/copain/frenchcropusage. The SPARQL EndPoint is http://ontology.inrae.fr/frenchcropusage/sparql. C3PO KB: The version 1.0 of the C3PO KB can be found in the gitlab repository https://gitlab.com/serre-des-savoirs/c3po-kb. The associated ontology can be found on the AgroPortal repository https://agroportal.lirmm.fr/ontologies/C3PO/?p=summary. The SPARQL EndPoint is https://graph.elzeard.co/sparql.

## Author contributions

BD supervised the building of C3PO and its KG and used within applications and wrote the manuscript. BD and FA built the ontology, the knowledge graph, and implemented the process with domain experts. BD, FA, and CR wrote the definitions of the ontology. CJ and CR helped in the modeling of some modules of the ontology and provided general knowledge engineering expertise. All authors contributed to the manuscript, read, and approved the final version.

## Footnotes

^1^ https://elzeard.co

^2^ https://www.ncbi.nlm.nih.gov/taxonomy

^3^ https://lab.allmende.io/valueflows

^4^ https://www.elzeard.co/ontologies/c3po/

^5^ https://www.drawio.com/

^6^ https://chowlk.linkeddata.es/

^7^ https://www.ontotext.com/products/ontotext-refine/

^8^ https://gitlab.com/serre-des-savoirs/c3po/-/blob/master/C3PO-Competency_question.xlsx

^9^ https://www.lexagri.com/products/basagri/

^10^ https://graphdb.ontotext.com/

^11^ https://www.elzeard.co/ontologies/c3po/

^12^ https://gitlab.com/serre-des-savoirs/c3po

^13^ https://www.datafoodconsortium.org/en/

^14^ https://agrohackathon2022.workshop.inrae.fr/

^15^ https://www.picleg.fr/Projets/Les-projets-en-cours/MESCLUN-DURAB

^16^ https://www.elzeard.co/ontologies/c3po/EN/EN_Plant.html

^17^ https://www.elzeard.co/ontologies/c3po/EN/EN_CM.html

^18^ https://www.elzeard.co/ontologies/c3po/EN/EN_CM.html
